# Accuracy of various criteria for lymph node staging in ductal adenocarcinoma of the pancreatic head by computed tomography and magnetic resonance imaging

**DOI:** 10.1186/s12957-020-01951-3

**Published:** 2020-08-18

**Authors:** Florian N. Loch, Patrick Asbach, Matthias Haas, Hendrik Seeliger, Katharina Beyer, Christian Schineis, Claudius E. Degro, Georgios A. Margonis, Martin E. Kreis, Carsten Kamphues

**Affiliations:** 1 Charité – Universitätsmedizin Berlin, corporate member of Freie Universität Berlin, Humboldt-Universität zu Berlin, and Berlin Institute of Health, Department of Surgery, Campus Benjamin Franklin, Hindenburgdamm 30, 12203 Berlin, Germany; 2 Charité – Universitätsmedizin Berlin, corporate member of Freie Universität Berlin, Humboldt-Universität zu Berlin, and Berlin Institute of Health, Department of Radiology, Campus Benjamin Franklin, Hindenburgdamm 30, 12203 Berlin, Germany; 3 The Johns Hopkins University School of Medicine, Department of Surgery, 600 N. Wolfe Street, Blalock 688, Baltimore, MD 21287 USA

**Keywords:** Ductal adenocarcinoma of the pancreatic head, Staging, Lymph nodes, Computed tomography, Magnetic resonance imaging, Cross-sectional imaging, Neoadjuvant therapy

## Abstract

**Background:**

Lymph node staging of ductal adenocarcinoma of the pancreatic head (PDAC) by cross-sectional imaging is limited. The aim of this study was to determine the diagnostic accuracy of expanded criteria in nodal staging in PDAC patients.

**Methods:**

Sixty-six patients with histologically confirmed PDAC that underwent primary surgery were included in this retrospective IRB-approved study. Cross-sectional imaging studies (CT and/or MRI) were evaluated by a radiologist blinded to histopathology. Number and size of lymph nodes were measured (short-axis diameter) and characterized in terms of expanded morphological criteria of border contour (spiculated, lobulated, and indistinct) and texture (homogeneous or inhomogeneous). Sensitivities and specificities were calculated with histopathology as a reference standard.

**Results:**

Forty-eight of 66 patients (80%) had histologically confirmed lymph node metastases (pN+). Sensitivity, specificity, and Youden’s Index for the criterion “size” were 44.2%, 82.4%, and 0.27; for “inhomogeneous signal intensity” 25.6%, 94.1%, and 0.20; and for “border contour” 62.7%, 52.9%, and 0.16, respectively. There was a significant association between the number of visible lymph nodes on preoperative CT and lymph node involvement (pN+, p = 0.031).

**Conclusion:**

Lymph node staging in PDAC is mainly limited due to low sensitivity for detection of metastatic disease. Using expanded morphological criteria instead of size did not improve regional nodal staging due to sensitivity remaining low. Combining specific criteria yields improved sensitivity with specificity and PPV remaining high.

## Background

Pancreatic cancer remains one of the most lethal malignancies being the fourth leading cause of cancer death in the USA and predicted to be the second leading cause of cancer death by 2020 [1]. The overall 5-year survival after diagnosis is 7% [2], and at the time of diagnosis, the main proportion of patients has advanced-stage disease leaving only 15–20% qualified for resective surgery [3]. Pancreatic cancer is located in the head of the pancreas in 75% of the cases [4]. Even after successful resective surgery in patients with cancer of the pancreatic head, the 5-year survival remains as low as 21% [5]. These data underline the importance of establishing multimodal therapeutic concepts for patients with pancreatic cancer as per other entities of abdominal cancer.

Apart from the potential to increase the resectability rate of pancreatic cancer by neoadjuvant therapy [6, 7], there is evidence that patients which are successfully downstaged from node-positive disease (cN1) to node-negative disease (ypN0) prior to surgery benefit in terms of higher 5-year survival rate [8]. This would qualify nodal involvement as a sufficient basis for indicating neoadjuvant therapy. Yet, even given advanced imaging technologies, identifying lymph node metastasis remains challenging. Consequently, the indication of a potentially effective neoadjuvant therapy (cN+) with side effects in lymph node-positive patients (cN+) is mainly based on unreliable clinical staging.

The established criterion for lymph node involvement in pancreatic cancer is size. Using the size underlies the assumption that tumor spread to regional lymph nodes leads to an enlargement of the respective lymph node. The usual cut-off value is a short-axis diameter of 10 mm [3, 9–12]. It has been shown though that lymph nodes of ≥ 10 mm are not seen more frequently in patients with histopathological lymph node involvement (pN+) [13]. In various other tumor entities, expanded morphological criteria such as texture and border contour of lymph nodes are used for the assessment of lymph node malignancy on both computed tomography (CT) and magnetic resonance (MR) imaging. This is utilized in order to improve the accuracy of lymph node staging [14–16]. By applying morphological criteria instead of Brown et al. size criterion alone, the sensitivity was improved from 42 to 85% and the specificity from 87 to 97% in lymph node staging of rectal cancer [16].

Thus, the aim of this study was to determine the accuracy of lymph node staging in patients with ductal adenocarcinoma of the pancreatic head by both computed tomography and magnetic resonance imaging using size and expanded morphological criteria.

## Material and methods

### Patients

**Fig. 1:**
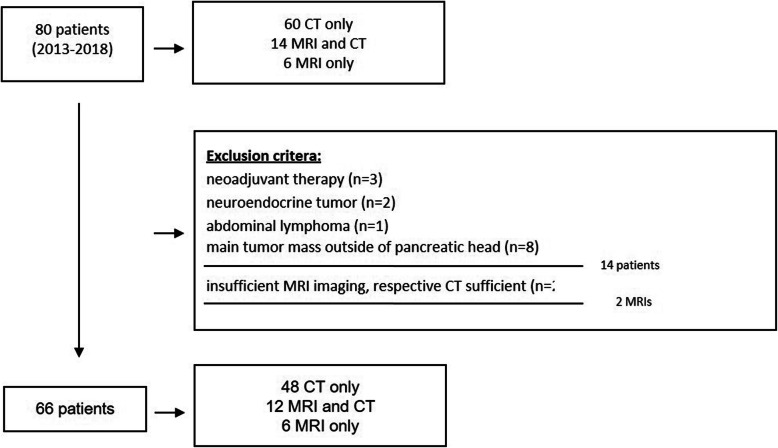
Flowchart of patient recruitment. The process of patient selection with the respective reasons for inclusion and exclusion is shown

In this retrospective single-center study approved by the local ethics committee, consecutive patients with histologically proven ductal adenocarcinoma of the pancreatic head that underwent primary surgery between February 2013 and November 2018 at the Department of Surgery, Campus Benjamin Franklin, Charité—University Medicine Berlin, Germany, were included. Patients were retrieved from the database of our pancreatic cancer center certified by the German Cancer Society (n = 80). Inclusion criteria were primary oncologic tumor resection and the presence of preoperative cross-sectional imaging of sufficient quality (see below). Exclusion criteria were neoadjuvant therapy, presence of a potential simultaneous cause of lymphadenopathy of the upper abdominal region (e.g. abdominal lymphoma, neuroendocrine tumor), and main tumor mass located outside the pancreatic head on histopathology. The process of patient selection with the respective reasons for inclusion and exclusion is shown in Fig. 1.

### Cross-sectional imaging

All images were retrospectively analyzed for the purpose of this study by a single abdominal radiologist, with more than 12 years of experience in staging of tumors of the visceral organs, blinded to the results of histopathology.

All cross-sectional imaging studies were assessed for sufficient image quality by the radiologist prior to commencement. For CT imaging, the minimum quality was defined as either thin-section CT (≤ 2 mm reconstructed slice thickness) or contrast-enhanced CT with a slice thickness of ≤ 5 mm. For MRI, minimum quality was defined as availability of an axial T2-weighted sequence with fat suppression (slice thickness ≤ 5 mm) in combination with a venous phase post-contrast 3D gradient-echo sequence (slice thickness ≤ 3 mm).

**Fig. 2:**
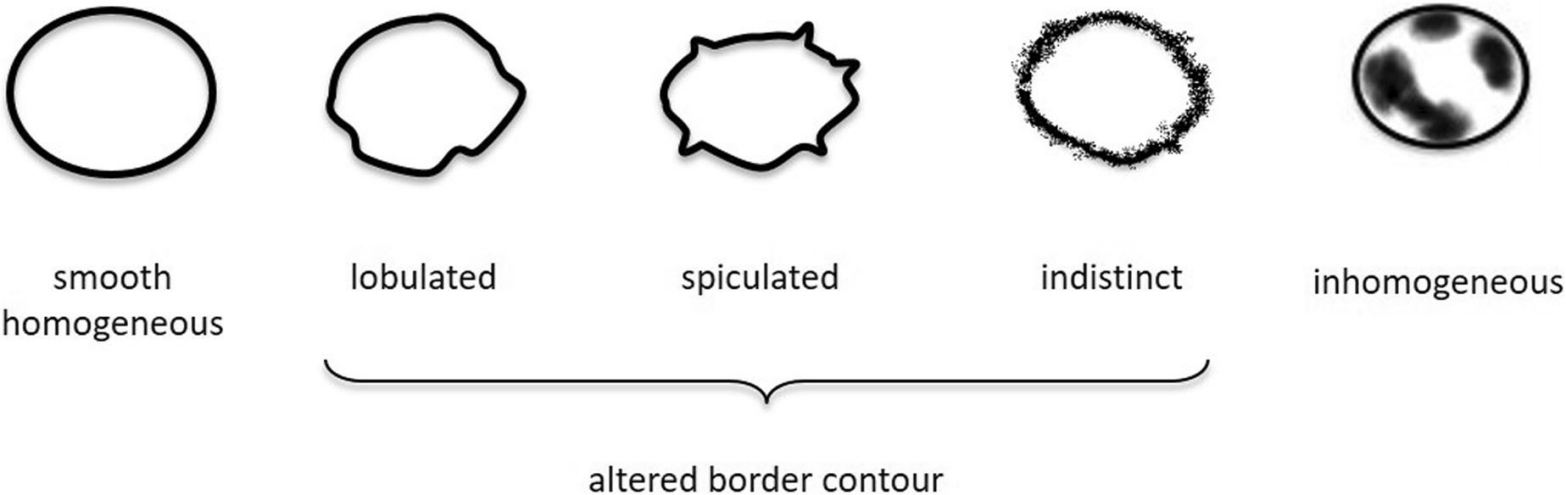
Morphological characterization of lymph nodes based on Kim et al. [16]. The morphological criteria for lymph node assessment used in this study are shown. Smooth and homogeneous lymph nodes were considered normal

For lymph node assessment, all visible regional lymph nodes in the field of view were recorded on a score chart and the total number of visible lymph nodes per patient was calculated. Then, for each patient, all lymph nodes were characterized in terms of size (long- and short-axis diameter in millimeters) and the expanded morphological criteria border contour (lobulated, spiculated, indistinct, or unaltered) and texture (homogeneous or inhomogeneous, Fig. 2 based on Kim et al. [17]).

Regional lymph nodes of the pancreas are defined as the following lymph node station numbers: 5, 6, 7, 8a, 8p, 9, 10, 11p, 11d, 12a, 12b, 12p,13a, 13b,14p,14d, 17a, 17b, and 18 [17]. In all cases in which a lymph node was not definitively regional, correlation with postoperative cross-sectional imaging was performed to assess whether the lymph node was resected or not. Only resected lymph nodes were analyzed in this study.

A second radiologist with more than 5 years of experience in staging of tumors of the visceral organs, also blinded to the results of histopathology, evaluated the CT examinations of a representative subgroup of 20 patients for evaluation of interobserver agreement.

### Surgery

All patients underwent primary, oncologic pylorus-preserving pancreaticoduodenectomy or Whipple procedure with complete lymphadenectomy of the regional lymph nodes mentioned above.

### Histopathology

For the study, the original histopathological reports using formalin-embedded surgical specimens were reviewed. Cancer of the pancreatic head was defined as a malignant tumor located within the pancreas to the right of the superior mesenteric vein and portal vein. Each patient with histologically proven lymph node metastases was classified as node-positive (pN+) regardless of the number of metastatic lymph nodes. Patients without any metastatic lymph nodes were classified as node-negative (pN-). The ratio of metastatic lymph nodes vs. the total number of retrieved lymph nodes was documented in the histopathological report (e.g., 0/14 or 3/23). Tumors were classified according to their respective TNM stage using the 8th Edition of TNM Classification of Malignant Tumors [18].

### Comparison of cross-sectional imaging and histopathology

Sensitivity, specificity, and positive predictive value of the nodal status using CT and MRI, with histopathology as a reference standard, were calculated for lymph node involvement using size and morphological criteria. Specifically, nodal involvement criteria were based on either size (short-axis diameter), altered border contour (lobulated, spiculated or indistinct), and inhomogeneous signal intensity (Fig. 2). CT and MRI examinations were considered node-positive (cN+), if at least one lymph node met one of the respective criteria used for involvement. If no lymph node with the respective criteria was seen on CT or MRI, then the examination was considered node-negative (cN-).

### Statistical analysis

Sensitivities, specificities, and positive predictive value (PPV) for the size criterion, and all morphological criteria were calculated for their respective cut-off values. An index summarizing the sensitivity and specificity for Youden’s Index was calculated (Sensitivity + Specificity − 1) [19]. The number of lymph nodes visible on CT and MR images in the group with (pN+) and without (pN-) nodal metastases was compared using the Mann-Whitney U test. When calculating the association between CT and MRI criteria and lymph node positivity, the χ2-test was used. Interobserver agreement was calculated using Cohen’s Kappa statistic. A p value of ≤ 0.05 was considered to indicate a statistically significant difference.

## Results

### Patients

Sixty-six patients were included in the study (Fig. 1) with the characteristics of the patients presented in Table 1. Sixty of these patients were staged by preoperative CT, twelve of which had additional staging by MRI, and six patients were staged by only MRI. In two patients, the MRI examinations were excluded due to insufficient imaging quality. Both patients had sufficient staging by CT and were therefore included in the study. Of the 66 patients, 10 patients received preoperative biliary drainage. Eight of them were staged by CT only and two by MRI only.

**Table 1:** Demographic data of patients with ductal adenocarcinoma of the pancreatic head undergoing primary tumor resection

Patients	n = 66
**Age**	
Median age (years)	73
Age range (years)	44–86
**Sex**	
Female	28 (42%)
Male	38 (58%)
**Cross-sectional imaging**	
CT only	48 (73%)
CT and MRI	12 (18%)
MRI only	6 (9%)
**Histopathological staging**	
pN+	48 (73%)
pN-	18 (27%)
pT1	5 (8%)
pT2	39 (59%)
pT3	22 (33%)

### Computed tomography (CT)

Lymph nodes were detected by CT in 96.7% (58/60) of the patients. The median number of visible lymph nodes was 5 (range 0–15). The smallest visible lymph node was 2.0 mm of size whereas the largest measured 18 mm (short-axis diameter). The mean time between CT and surgery was 7 days with a median of 6 days (range 1–43). The slice thickness in 61 of the 66 CT examinations (92.4%) was 3 mm or less. In five CT examinations (7.6%), slice thickness was 5 mm.

### Size criterion for lymph node involvement on preoperative CT

**Fig. 3:**
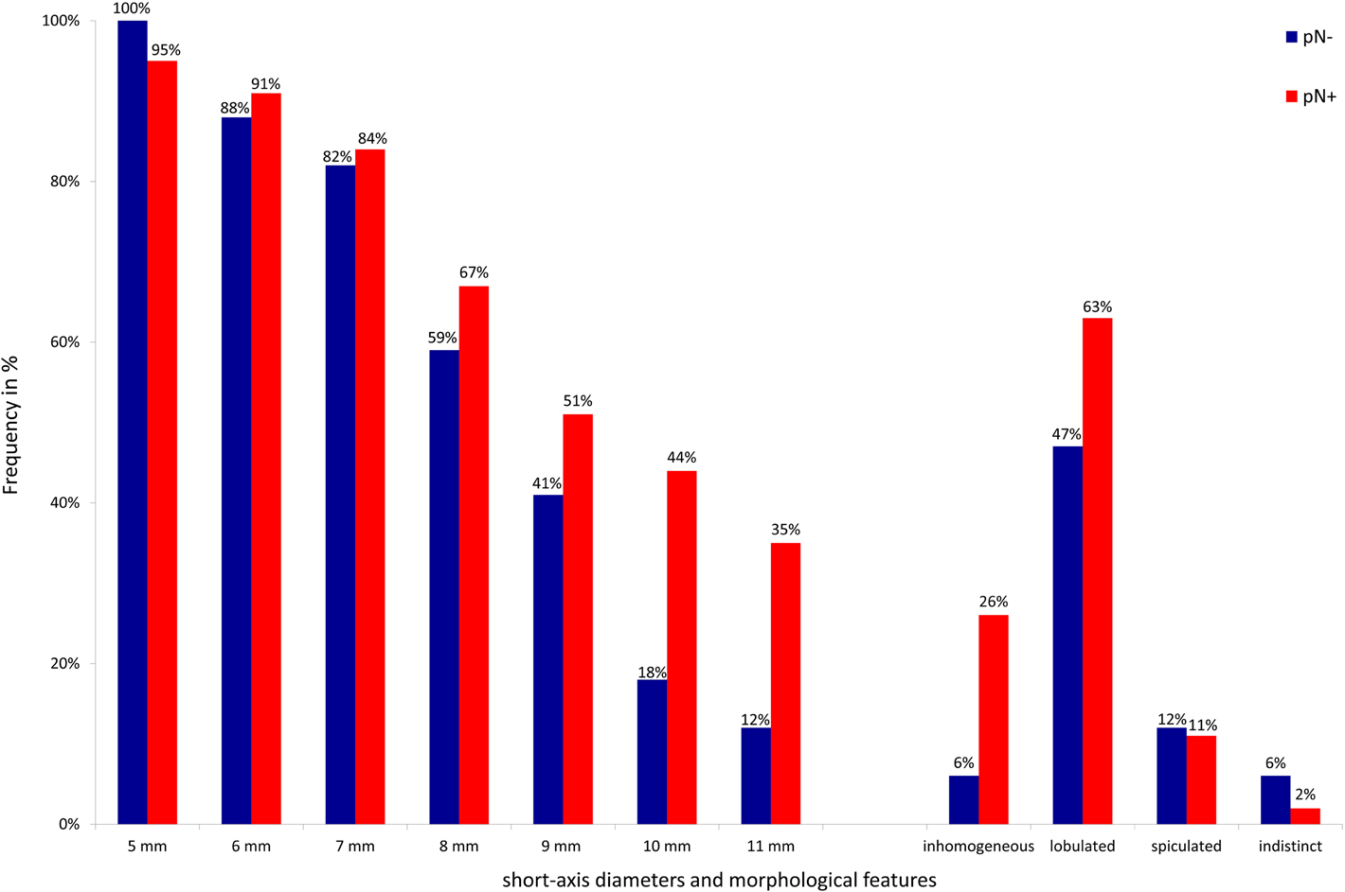
Graph showing lymph node size and morphological criteria of lymph node-positive and -negative patients. Frequency of regional lymph nodes of the pancreatic head in percent (x-axis) with different short-axis diameters and morphological features (y-axis) in patients with (pN+, red bars) or without histologically proven lymph node metastases (pN-, blue bars) on preoperative CT imaging

Figure 3 shows the percentage of patients with (pN+) and without (pN-) lymph node metastases in which a lymph node of the respective size was visible (5–11 mm). In Table 2, sensitivity, specificity, and Youden’s Index are presented for the respective cut-off values. Lymph nodes of small and medium size (5–9 mm) were visible in patients with (51–95%; 22–41/43, pN+) and without lymph node metastases (41–100%; 7–17/17, pN-) in even frequency. Large lymph nodes (10–11 mm) were seen more frequently in the lymph node-positive group (35–44%; 15–19/43, pN+) than in the lymph node-negative group (12–18%; 2–3/17, pN-). The maximum value of Youden’s Index for the size criterion was J = 0.27 (95% CI; 0.00, 0.45) when a cut-off value of 10 mm was applied, yielding a sensitivity of 44% and specificity of 82%. Additionally, the presence of lymph nodes greater than 10 mm on preoperative CT, and the histopathological confirmation of a lymph node metastasis (pN+), showed a trend towards significance (p = 0.076).

**Table 2:** Sensitivity, specificity, PPV, and Youden’s Index for cut-off values and morphological criteria by CT and MRI

	Sensitivity	Specificity	PPV	Youden’s Index
**CT**				
**Size, cut-off value**				
4 mm	95.3%	0%	70.7%	− 0.05
5 mm	95.3%	0%	70.7%	− 0.05
6 mm	90.7%	11.7%	72.2%	− 0.02
7 mm	83.7%	17.6%	72.0%	0.01
8 mm	67.4%	41.2%	74.4%	0.08
9 mm	51.1%	58.8%	75.9%	0.10
10 mm	**44.2%**	**82.4%**	**86.4%**	**0.27**
11 mm	34.9%	88.2%	88.2%	0.23
**Morphological criterion**				
Lobulated	62.7%	52.9%	77.1%	0.16
Spiculated	11.6%	88.2%	71.4%	− 0.00
Indistinct	2.3%	94.1%	50.0%	− 0.04
**Inhomogeneous**	**25.6%**	**94.1%**	**91.7%**	**0.20**
**MRI**				
**Size, cut-off value**				
7 mm	75.0%	0%	60.0%	− 0.25
**8 mm**	**58.3%**	**83.3%**	**87.5%**	**0.42**
9 mm	58.3%	66.7%	77.8%	0.25
**10 mm**	**58.3%**	**83.3%**	**87.5%**	**0.42**
11 mm	25.0%	83.3%	75.0%	0.08
12 mm	16.7%	83.3%	66.7%	0.00
13 mm	16.7%	83.3%	66.7%	0.00
14 mm	8.3%	83.3%	50.0%	− 0.08
15 mm	8.3%	83.3%	50.0%	− 0.08
**Morphological criterion**				
Lobulated	16.7%	66.7%	50.%	− 0.17
Spiculated	8.3%	100%	35.3%	0.08
Indistinct	Not visible			
Inhomogeneous	Not visible			

### Expanded morphological criteria for lymph node involvement on preoperative CT

Figure 3 shows the percentage of patients with (pN+) and (pN-) without lymph node metastases in which a lymph node of the respective morphological criterion was visible and Table 2 shows the sensitivity, specificity, and Youden’s Index of the respective criterion.

Lymph nodes of lobulated border contour were visible with a similar frequency in patients with (63%; 27/60, pN+) and without lymph node metastases (47%; 7/17, pN-). Lymph nodes of spiculated or indistinct border contour were only occasionally detected in both groups (11%; 5/43 vs. 2%; 1/43 in the lymph node-positive group (pN+) and 12%; 2/17 vs. 12% 2/17 in the lymph node-negative group (pN-)).

Lymph nodes of inhomogeneous signal intensity were detected in only one patient of the lymph node-negative group (6%; 1/17, pN-) and more frequently in patients of the lymph node-positive group (26%; 11/43, pN+) resulting in the maximum value of Youden’s Index for the morphological criteria J = 0.20 (95% CI; 0.04, 0.35), consisting of a sensitivity of 26% and a specificity of 94%. The PPV was 91.7%.

### Comparison of size with expanded morphological criteria

**Fig. 4:**
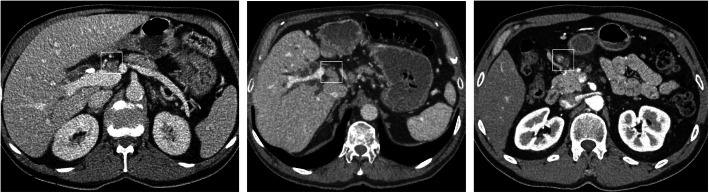
CT images of patients with and without lymph node metastases. **a** Patient with enlarged suspicious lymph node adjacent to the portal vein and hepatic artery who had no lymph node metastases on pathology. **b** Patient with enlarged suspicious lymph node adjacent to the hepatic artery who had lymph node metastases on pathology. **c** Patient with multiple suspicious lymph nodes based on size and inhomogeneity who had lymph node metastases on pathology

The maximum value of the Youden’s Index of the “size” criterion was J = 0.27 (95% CI; 0.00, 0.45; cut-off 10 mm) which is not inferior to the maximum value of the morphological criteria J = 0.20 (95% CI; 0.04, 0.35; inhomogeneous signal intensity). Figure 4 displays respective CT images of patients with and without lymph node metastases.

### Number of visible lymph nodes

There was a significant association between number of visible lymph nodes seen on preoperative CT and histopathological lymph node involvement (pN+, p = 0.031).

Seven or more lymph nodes were seen on preoperative CT in 32.6% (14/43) of patients with lymph node metastases (pN+) and in 5.9% (1/17) of patients without lymph node metastasis (pN-, p = 0.046). This resulted in a specificity of 94.1%, Youden’s Index of 0.27, and PPV of 93.3%.

### Combination of number, size, and expanded morphologic criteria on preoperative CT

Combining the size criterion and the morphological criterion with the respective highest Youden’s Index (cut-off 10 mm and “inhomogeneous signal intensity”) and the criterion “visible lymph nodes n ≥ 7” was significantly associated with nodal metastases (pN+, p = 0.004). For this combined criterion, specificity was 82%, sensitivity 61%, PPV 90%, and Youden’s Index 0.43 (95% CI; 0.15, 0.60).

### Interobserver agreement

Interobserver agreement was calculated for 20 patients (pN+, 75% pN+ vs. 25% pN-) for the criteria size, morphology, and number of visible lymph nodes with the respective highest Youden’s Index. Interobserver agreement was substantial for size (10 mm cut-off, κ = 0.8, p = 0.001), moderate for the presence of seven or more lymph nodes (κ = 0.571, p = 0.032), and fair for the morphological criterion inhomogeneous signal intensity (κ = 0.306, p = 0.202).

### Magnetic resonance imaging (MRI)

Lymph nodes were detected in 88.9% (16/18) of patients on preoperative MRI. The median number of visible lymph nodes was 3 (range 0–6). There was no significant association between number of visible lymph nodes seen on preoperative MRI and histopathological lymph node involvement (pN+, p = 0.682).

The smallest visible lymph node was 3.0 mm of size, whereas the largest measured 16 mm (short-axis diameter). The mean time between MRI and surgery was 11 days with a median of 7 days (range 1–38).

The cut-off values of the highest diagnostic value were 8 mm or 10 mm for the “size” criterion (sensitivity 58.3%, specificity 83.3%, Youden’s Index = 0.42). The presence of a lymph node of these sizes was not associated with lymph node metastases (pN+, p = 0.152). Lobulated and spiculated lymph nodes were only seen in a few patients (n = 4 and n = 1), and indistinct and inhomogeneous lymph nodes were not seen at all (Table 2).

## Discussion

In this retrospective single-center study on lymph node staging by CT in ductal adenocarcinoma of the pancreatic head, we could show that the morphologic criteria “inhomogeneous signal intensity” and “size” are specific for regional nodal metastatic disease. Replacing the size criterion by morphologic criteria, however, did not improve diagnostic accuracy due to sensitivity remaining low. Combining specific criteria yields improved sensitivity with specificity remaining high.

By CT, lymph nodes of 4–9 mm in short-axis diameter were seen just as often in patients with and without lymph node metastases resulting in poor discrimination. Larger lymph nodes (> 9 mm) had a higher prevalence in the lymph node-positive group leading to high specificity. However, these lymph nodes (10 mm or 11 mm) were seen infrequently resulting in a rather low sensitivity. The maximum value of the Youden’s Index for the size criterion of 0.27 was achieved when a cut-off value of 10 mm was applied, consisting of a specificity of 82.4% and sensitivity of 44.2%, yielding a PPV of 84.6%.

As for morphologic criteria, lymph nodes of lobulated border contour were seen in about half of the patients of both groups (pN+, 63% and pN-, 47%), and therefore is a criterion that is not suitable to differentiate between the groups. Lymph nodes of spiculated and indistinct border contour were seen in few cases in both patient groups only (pN+ 11% and 2% versus pN- 12% and 6%) making them poor diagnostic criteria. However, lymph nodes of inhomogeneous signal intensity were visible in 26% of patients with lymph node metastases (pN+) and only in 6% of the patients without lymph node metastases (pN-), resulting in a Youden’s Index of 0.20, which was the maximum value for the morphological criteria, and a PPV of 91.7%.

Ideally, a good discriminator for nodal metastases is negative in patients without nodal involvement and positive for tumors with lymph node metastases. In our study, each criterion, i.e., size as well as different morphological features, only met one of these prerequisites. The size criterion (10 mm) as well as the presence of a lymph node of inhomogeneous signal intensity as morphological criterion turned out to be negative in patients without nodal involvement (pN-) and therefore highly specific. Yet, lymph nodes of the respective characteristic were not positive in a sufficiently high number of tumors with lymph node metastases (pN+) to reach high levels of sensitivity and consequently did not have a significant diagnostic value.

The maximum value of the Youden’s Index for the size criterion was 0.27 when a cut-off value of 10 mm was applied and 0.20 for the morphological criteria, when the criterion “inhomogeneous signal intensity” was used, showing that morphologic criteria do not yield in higher diagnostic value than lymph node size in adenocarcinoma of the pancreatic head (PDAC) patients. This is contrary to the findings of Brown et al. in rectal cancer [16]. One reason might be that Brown et al. used MRI to assess morphologic criteria which has a higher soft tissue contrast compared to CT which was used in most patients in our study.

Interestingly, we could show that with preoperative CT, the presence of seven or more lymph nodes was seen more often in patients with lymph node metastasis (pN+) than in those without metastasis (pN-, p = 0.046). When applying this as a sole criterion (cN) for lymph node metastasis (pN), this led to a sensitivity of 32.6%, a specificity of 94.1%, PPV of 93.3%, and Youden’s Index of 0.27.

In diagnostic test analysis, criteria can be combined in mainly two ways: sensitive criteria can be taken together to improve specificity or specific criteria can be accumulated to improve sensitivity. When combining the highly specific criteria size (cut-off value 10 mm), inhomogeneous signal intensity, and number of visible lymph nodes n ≥ 7, a highly significant association with nodal metastases (pN+, p = 0.004) was found. Consequently, the CT examination was considered node-positive (cN+) when at least one of these criteria was met. The application of this criterion improved the sensitivity to 60% with a remaining specificity of 82% and PPV 90% resulting in an also improved Youden’s Index of 0.43.

The results of the MRI examinations must be viewed in a rather descriptive manner since the sample size was limited (n = 18). Lymph nodes were detected in the majority (88.9%) of examinations generally allowing the evaluation of lymph nodes by MRI as well. Upper abdominal MRI generally has a lower spatial resolution, but a higher soft tissue contrast compared to CT. For the size criterion a cut-off value of 8 mm or 10 mm led to the best diagnostic results (sensitivity 58.3%, specificity 83.3%, Youden’s Index = 0.42). Lymph nodes of abnormal morphological criteria were seen in only very few patients (Table 2).

The main limitation of this study is the retrospective study design in which a node-by-node comparison of cross-sectional imaging with histopathology was not possible. This was of minor importance, though, since low sensitivity was the main factor that led to compromised diagnostic performance in our study. We were also able to correlate with postoperative cross-sectional imaging in all cases in which it was unclear whether a lymph node had been resected during surgery or not. Also, in our cohort, only patients who had subsequent surgery were included, presuming lower tumor stage as compared to the average patient who undergoes imaging for presurgical workup.

The strength of our single-center study is reinforced by a defined number of surgeons, a high standardization of the CT technique, and an experienced radiologist who performed the analysis.

The results of our study are consistent with recent and initial data demonstrating that clinical staging, by low sensitivity, underestimates histopathological lymph node involvement (pN+) [6, 20–22]. However, by adding the criterion “inhomogeneous signal intensity” and “number of visible lymph nodes” to the size criterion, we were able to increase the sensitivity to 60% in comparison to previous findings (14%, Roche et al.; 37% Nanashima et al.; and 46.2%, Cao et al.) with specificity remaining sufficient.

An additional imaging modality that has shown the potential to improve the sensitivity of detecting metastatic disease is positron emission tomography-computed tomography (PET/CT) [23]. However, a beneficial role of PET/CT in locoregional nodal staging could not be established to date. The majority of initial as well as recent studies show very limited sensitivities for nodal status between 10 and 61% [24–29]. The PET-PANC study evaluated the incremental diagnostic accuracy and impact of PET/CT in addition to multidetector CT in patients with suspected pancreatic cancer in a prospective multicenter study that included 550 patients. In this study, significantly more patients with stage IIb disease (pN+) were correctly staged by PET/CT than by multidetector CT (p = 0.002), but this only led to a moderate sensitivity of 38% for PET/CT versus 22% for multidetector CT [30].

Endoscopic ultrasonography (EUS) is a well-established diagnostic procedure in pancreatic cancer with the benefit of a dynamic diagnostic examination that allows fine-needle aspiration for cytologic diagnosis. Two meta-analyses evaluating diagnostic accuracy of EUS for locoregional nodal staging the pooled sensitivities and specificities were 0.62 and 0.74 (Li et al 2014, 14 studies, n = 516 patients) [31] and 69% and 81% (Nawaz et al., 16 studies, n = 512 patients) [32]. Advanced techniques such as contrast-enhanced EUS (CH-EUS) and EUS elastography are currently in evaluation [33].

To date, CT remains the standard staging imaging modality recommended by NCCN guidelines for locoregional staging of pancreatic cancer [34]. Neither PET/CT nor EUS yields reliable diagnostic accuracy for nodal staging.

An advantageous effect on resectability and overall survival (OS) in unresectable cases (including both borderline resectable and unresectable) of PDAC by multimodality therapy including neoadjuvant therapy has already been described in several studies [35].

The benefit of neoadjuvant therapy in cases of primarily resectable disease at diagnosis is yet less revealed. Several phase II trials showed that patients who completed neoadjuvant chemoradiation without progressive disease at restaging had a higher chance of achieving R0 resection and, consequently, higher median and OS when compared to historical data [36]. As seen in other tumor entities, a potential benefit of neoadjuvant therapy on the basis of positive nodal status (cN+) is strongly implied. Cao et al. found that the 38% of patients that were successfully downstaged from node-positive disease (cN1) to node-negative disease (ypN0) by neoadjuvant therapy benefit in terms of higher rates of 5-year survival (ypN0 27.2% vs ypN1 12.3%, p < 0.001) [8]. This is consistent with the findings of Portuondo et al. (5-year survival ypN0 12.4% vs. ypN1 6.0%, p < 0.0001) [37]. The NCCN guidelines for pancreatic adenocarcinoma appreciates these results by stating that consideration can be given to neoadjuvant therapy for selected patients with resectable tumor but poor prognostic features such as large regional lymph nodes, markedly elevated CA 19-9, large primary tumors, extreme pain, and excessive weight loss [34]. Further clarification on this matter is expected to come from the ongoing NEONAX trial (NCT02047513), a phase II study comparing neoadjuvant plus adjuvant with only adjuvant nab-paclitaxel plus gemcitabine therapy for resectable pancreatic cancer. The ongoing phase III NEOPA trial (NCT01900327) compares neoadjuvant chemoradiotherapy with upfront surgery of resectable pancreatic head cancer. A subgroup analysis in terms of nodal status would present reliable data.

Given the suggested benefit of neoadjuvant therapy based on lymph node staging, there is an urgent need to find criteria and modalities to further improve the diagnostic value of lymph node staging by pretherapeutic cross-sectional imaging in patients with ductal adenocarcinoma of the pancreatic head. To date, none of the existing modalities and criteria accomplishes reliable nodal staging. Larger, prospective studies are ongoing and necessary to get a more precise idea of the prognostic advantage of neoadjuvant therapy in patients with regional lymph node metastasis (cN+) of PDAC in pretherapeutic staging.

## Conclusions

Lymph node staging in PDAC patients when using CT morphological criteria such as border contour or homogeneity compared to diameter cut-off values does not lead to reliable diagnostic value. Diagnostic accuracy is limited due to low sensitivity for detection of metastatic disease. Combining specific criteria yields improved sensitivity with specificity and PPV remaining high. These results suggest an attentive interpretation of the results of pretherapeutic lymph node staging, particularly in cases in which lymph node metastases are absent.

## Data Availability

The datasets used during the current study are available from the corresponding author on reasonable request.

## Abbreviations

PDAC: Adenocarcinoma of the pancreatic head
CT: Computed tomography
MRI: Magnetic resonance imaging
pN+: Histopathologically involved lymph nodes
pN-: Histopathologically no involved lymph nodes
cN+: Clinically involved lymph nodes
cN-: Clinically no involved lymph nodes
PPV: Positive predictive value
PET/CT: Positron emission tomography-computed tomography
EUS: Endoscopic ultrasonography
CH-EUS: Contrast-enhanced endoscopic ultrasonography
OS: overall survival
